# Three cases of cutaneous blastomycosis in immunocompetent patients

**DOI:** 10.1016/j.jdcr.2025.05.044

**Published:** 2025-07-07

**Authors:** Jane Brown, Carolyn Ziemer, Diana McShane, Jayson Miedema, Rachel C. Blasiak

**Affiliations:** a University of North Carolina School of Medicine, Chapel Hill, North Carolina; b Department of Dermatology, University of North Carolina School of Medicine, Chapel Hill, North Carolina

**Keywords:** Blastomycoses, blastomycosis

## Introduction

Blastomycosis is a rare systemic fungal infection endemic to the Ohio River Valley, Great Lakes, and the Southwest, often misdiagnosed or missed, especially in immunocompetent patients. Retrospective cohort studies in hospital systems where *Blastomycoses* is endemic report that on average 30% of patients diagnosed with blastomycosis are immunocompromised.^1^^,^^2^ The incidence in immunocompromised patients, particularly those with solid-organ transplants, is nearly 20-fold higher than in the general population.^3^ The most common risk factor is residence in an endemic area. As inhalation of infectious conidia is the primary route of transmission, disruption of wet soil or organic matter inhabited by *Blastomycoses* species poses the greatest risk of infection. Proximity to construction, hunting/fishing, outdoor activities or occupation, and proximity to bodies of freshwater are major exposure risks.^1^ Severe disease risk factors include underlying pulmonary multilobar disease, obesity, diabetes mellitus, and immunosuppression.^2^ Although blastomycosis responds well to treatment with antifungal therapy, case fatality can be as high as 78% without treatment.^4^ Rates of blastomycosis-associated hospitalizations are increasing in parts of the United States, including the Midwest and South. Additionally, disease occurrence has increased in areas beyond its endemic region, including other parts of the world like sub-Saharan Africa, suggesting an expanding geographic distribution.^5^

The means of transmission of this fungus is largely unknown, as most attempts to isolate it have been unsuccessful.^6^ Pulmonary infection is the most common presentation following inhalation of aerosolized spores. However, presentations range from asymptomatic to fulminant and are often nonspecific. Dermatologic spread is the most common form of extrapulmonary disease.^3^ Case studies show that cutaneous manifestations are crucial for diagnosing previously unknown disseminated blastomycosis.

Three unusual presentations of blastomycosis in immunocompetent individuals illustrate the importance of dermatological evaluation and identification of cutaneous disease in diagnosing disseminated blastomycosis.

## Case presentations

### Case 1

**Fig 1:**
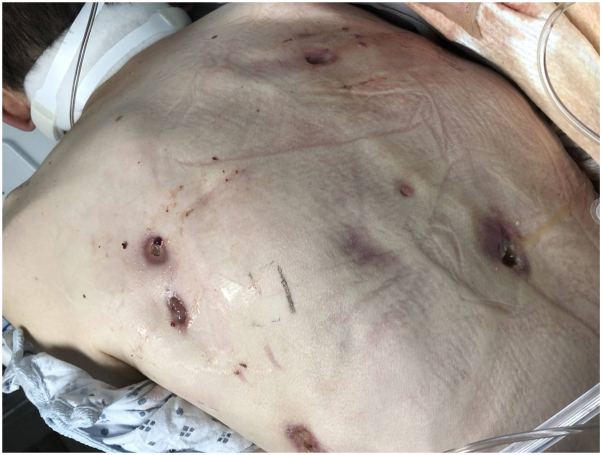
Case 1: Tunneling sinus tracts with surrounding erythema and purulent drainage to the back.

**Fig 2:**
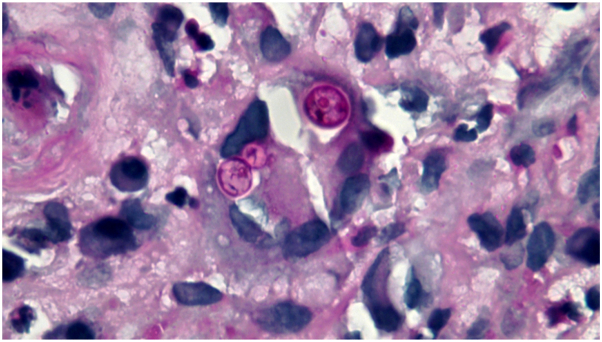
Case 1: Broad-based budding 10-12 micrometer yeasts, 1000× oil PAS. *PAS*, Periodic acid-Schiff.

A 19-year-old previously healthy male from an endemic region of western North Carolina presented in the emergency department with acute respiratory failure and septic shock from influenza A infection. Patient history included a brown recluse spider bite to mid-back 3 years ago, still in the process of healing, and other nonhealing boils on his back assumed to be hidradenitis. The patient was intubated and started on vancomycin and cefepime. A punch biopsy of the draining lesion on the patient’s back was obtained for dermatopathology and tissue culture. The initial read of this biopsy showed an ulcer with superficial granulation tissue and underlying edematous dermal scar, similar to hidradenitis suppurativa. Surface skin aerobic swab cultures grew *Streptococcus pyogenes*. The patient initially improved with antibiotics and was extubated the following day. Two days later he acutely decompensated and was reintubated. Further analysis revealed a bronchopleural fistula with air tracking through the spinal cord out through the back abscess, a right pleural cutaneous fistula, and multilevel lytic spine and posterior rib lesions (Fig 1). The patient’s coinciding weight loss raised suspicion for an immunodeficiency or malignancy. Five days after collection, skin biopsy cultures grew *Blastomycoses* species and periodic acid-Schiff (PAS) stain of the back punch biopsy showed rare round PAS positive bodies with a cell wall (Fig 2). The patient was also found to have elevated urine *Histoplasma* antigen, which is known to have cross-reactivity with *Blastomycoses*. The patient was diagnosed with disseminated blastomycosis with skin, pulmonary, and bone involvement. The patient was started on liposomal amphotericin B, with a plan for step-down azole therapy with itraconazole upon clinical improvement. Underlying immunodeficiency, such as hyper immunoglobulin (Ig) E and granulocyte disorders (chronic granulomatous disease) was ruled out with normal IgE, neutrophil function, and lymphocyte markers. However, the patient was found to have a severe vitamin A deficiency of less than 5 mcg/dL. The patient was discharged back to his local hospital after 51 days for continued treatment with itraconazole. The patient has continued itraconazole since diagnosis, completing nearly a year of treatment on last encounter. Other than a comorbidity of bilateral foot drop, the patient is doing well with completely healed skin lesions on his back and axillary region and no systemic symptoms or new lesions.

### Case 2

**Fig 3:**
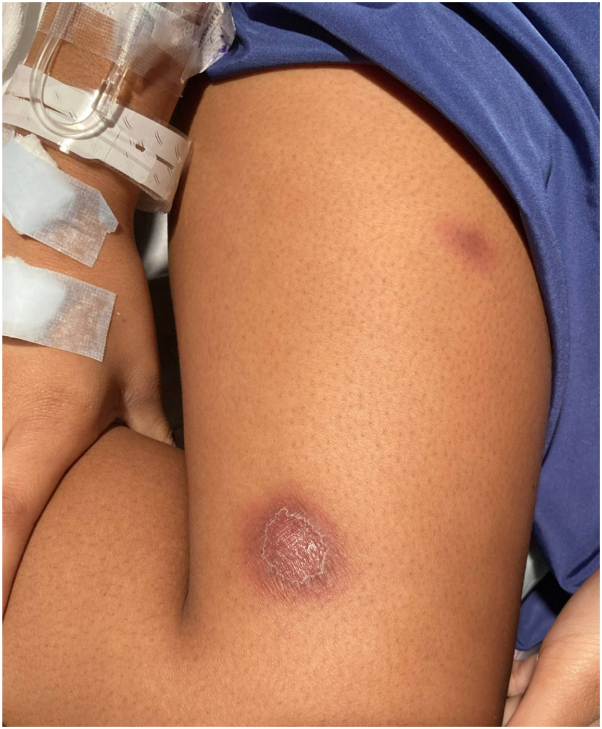
Case 2: Violaceous nodules with peripheral erythema to thigh.

A 16-year-old female was admitted for nonhealing skin lesions on her thigh thought to be associated with a fall onto a mat during cheerleading practice, at which time there were no appreciable open wounds (Fig 3). The lesions were initially diagnosed as an infected hematoma and treated with Keflex. The patient returned to the hospital 7 days later with multiple new subcutaneous nodules on all extremities, some spontaneously draining and painful. This coincided with a 22-pound unintentional weight loss and fever. Magnetic resonance imaging showed multiple superficial abscesses, cellulitis, and myositis of the subcutaneous soft tissues of both thighs. Two aerobic blood cultures taken 10 days apart were negative, as well as antinuclear antibody, double-stranded DNA, urine gonorrhea/chlamydia, HIV, and antistreptolysin O titer testing. Initial laboratory evaluation was significant for elevated C-reactive protein and erythrocyte sedimentation rate, low hemoglobin, and increased platelets. The patient’s white blood cell count was normal. The patient was initially started on vancomycin and ceftriaxone, and then transferred to our medical center where clindamycin intravenous (IV) monotherapy was begun. Following a dermatology consult, punch biopsies of a new left upper extremity nodule were obtained for pathology and tissue aerobic, fungal, and atypical mycobacterial cultures. The differential diagnosis at the time included infectious bacterial or fungal etiology, neutrophilic dermatosis, and panniculitis, with high suspicion for an underlying systemic process given the elevated inflammatory markers, weight loss, fevers, and joint pain. The patient’s fungal cultures began growing mold on day 5, but fungal organisms were not identified by tissue pathology despite multiple reviews by dermatopathology. On day 12, the fungal culture was identified as *Blastomyces* despite negative urinary *Blastomyces* antigen obtained 2 days prior to skin biopsies. At the time of diagnosis, chest X-ray and computed tomography scan showed pulmonary and bone involvement. Despite significant autoimmune history in her family, underlying immunodeficiency was ruled out with mu;titime point reads of IgG, IgM, and IgA antibodies; lymphocyte markers; neutrophil function; complete blood count; and complete metabolic panels. The patient was begun on itraconazole 200 mg twice daily with monitoring of serum levels of medication, blood counts, and complete metabolic panels. Due to bony involvement, she is currently completing a 12-month treatment course and is responding well to therapy.

### Case 3

**Fig 4:**
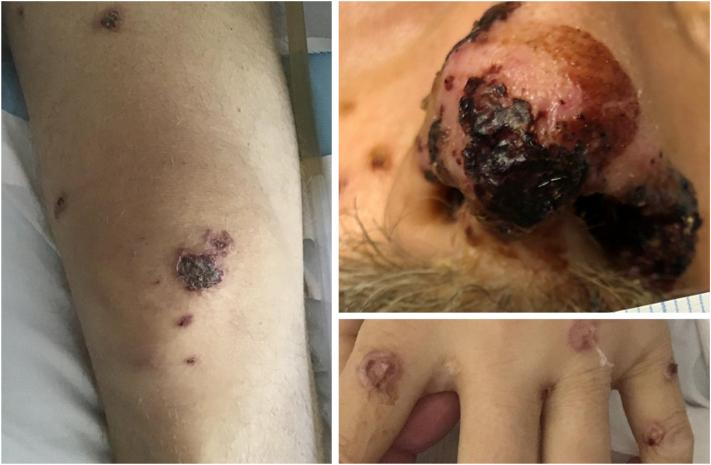
Case 3: Scattered pink papules and plaques with hemorrhagic crusting to hand, thigh, and glans penis.

**Fig 5:**
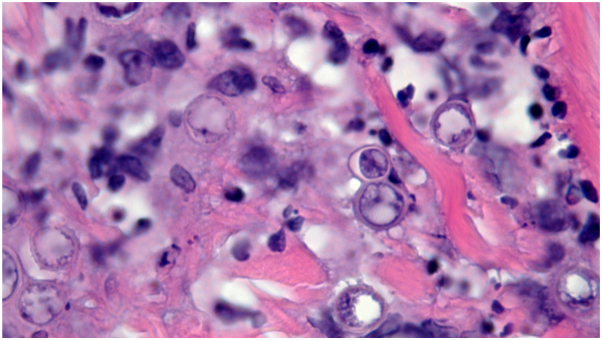
Case 3: Broad-based budding 10-12 micrometer yeasts, 1000× oil H&E. *H&E*, Hematoxylin and eosin.

A 52-year-old male from an endemic region of western North Carolina with type 2 diabetes mellitus was admitted to a local medical center in acute hypoxic respiratory failure, thought to be community-acquired pneumonia. He had diffuse scattered skin lesions with purulent drainage on his face, arms, legs, and left foot (Fig 4) and a skin biopsy was performed. Per family report, the patient had lost approximately 60 pounds over the 2 years prior to presentation. Sarcoidosis was suspected based on computed tomography scan and lack of evidence of infection on bronchoscopy. He was initiated on prednisone and amoxicillin/clavulanate and discharged. The patient returned to the emergency department a week later and was found to have innumerable centrilobular nodules on chest imaging with clinical concern for worsening sarcoidosis. The dermatopathology analysis of skin biopsy results returned on the ninth day with changes concerning for blastomycosis (Fig 5) and he was started on liposomal amphotericin B. The patient was transferred to our quaternary medical center due to septic shock, acute renal failure, and respiratory decompensation requiring intubation. The patient was started on IV amphotericin B at a limited 5 mg/kg/day dose with close monitoring given his acute kidney injury. There was concern for ocular and bony involvement of his disseminated fungal infection. Although skin biopsies demonstrated many organisms on hematoxylin and eosin staining consistent with disseminated blastomycosis, skin and tracheal aspirate cultures did not speciate *Blastomyces dermatitidis* until 2 months later. The patient completed a 6-week course of IV amphotericin at The University of North Carolina and began de-escalation to itraconazole therapy before being transferred to a local acute care hospital with instructions to continue itraconazole load. At the time of discharge, his hospital problems, including acute renal failure, metabolic acidosis, shock, and acute chronic respiratory failure with hypoxia requiring prolonged ventilation, remained unresolved. Three days after his initial discharge, the patient was readmitted to our quaternary medical center for oliguria and worsening renal function. Itraconazole had not been immediately available upon his transfer to the local acute care center and amphotericin had to be discontinued due to worsening renal function. The patient’s renal function improved upon back-transfer with continuous renal replacement therapy and hemi-dialysis. His acute respiratory distress syndrome and osteomyelitis resolved prior to discharge to a local long-term acute care hospital. The patient was lost to follow-up after discharge and expired a month and a half later.

## Discussion

Blastomycosis is a systemic fungal infection, primarily caused by the fungi *Blastomyces dermatitidis* in North America.^3^ The primary site of infection is the lungs, where inhaled spores undergo a dimorphic switch to yeast that evade immune defenses.^1^ Cutaneous blastomycosis is the most common extrapulmonary manifestation. Presentations are commonly described as ulcerative plaques or verrucous lesions, often on the head and extremities; however, as these cases demonstrate, the cutaneous manifestations of this disease are more diverse. These lesions can mimic other common diagnoses, particularly basal and squamous cell carcinoma and pyoderma gangrenosum.^7^ Immunosuppression is a risk factor for blastomycosis and is associated with severity of disease.^2^

In this case series, all patients were initially misdiagnosed and treated for bacterial infection. They were later diagnosed with disseminated blastomycosis after skin biopsies revealed characteristic pathology or fungal culture grew *Blastomycoses*.

Difficulty recognizing blastomycosis due to varied disease presentation and under-recognition of the disease are cited reasons for delayed diagnosis. The disease can present asymptomatically, with mild chronic pneumonia, or as acute respiratory distress syndrome. The disease may mimic community-acquired pneumonia, tuberculosis, or cancer.^8^ Pulmonary dissemination of blastomycosis is most common, commonly showing alveolar infiltrates, mass lesions, or fibronodular interstitial infiltrates on radiology. Up to two-thirds of patients experience extrapulmonary disease, mostly to the skin, bones, and genitourinary system. Cutaneous lesions without clinically active pulmonary disease are common, with nonpulmonary manifestations occurring in 25% to 40% of infected people.^5^

The manifestations of disease in these patients all included skin lesions that were misattributed to an exogenous cause (injury, spider bite) or systemic inflammatory disease (sarcoidosis). In these cases, diagnosis was delayed due to difficulty culturing the fungus, false-negative *Blastomycoses* antigen urine testing, and cross-reactivity with *Histoplasma* antigen on urine analysis. Once mold growth was detected in cultures, it took several days to confirm *Blastomycoses*. It took 6 days for culture to grow mold in Case 1 and 5 days in Case 2. Initial dermatopathology (performed the day after the patient’s back punch biopsy) misdiagnosed Case 1 as an ulcer with granulation tissue and edematous scarring. The detection of mold growth on day 6 and subsequent molecular testing was what suggested *Blastomyces* species. Blastomycosis was confirmed 7 days later by dermatopathology, with rereview of infectious staining revealing round PAS-positive bodies characteristic of *Blastomycoses* yeast forms. Once mold was detected in culture in Case 2, it took several attempts by dermatopathology to identify fungal organisms, blastomycosis diagnosis being made by fungal culture 7 days later. In Case 3, diagnosis of blastomycosis from skin biopsy analysis occurred 9 days after punch biopsies of the lesions were taken.

Despite taking days to weeks, microbiologic culture of specimens remains the standard for diagnosis. Direct microscopic visualization of organisms, that is, the characteristic yeast, in cytologic and histologic specimens is most commonly used for rapid blastomycosis diagnosis.^8^ However, fungal culture should be paired with histopathology to ensure accurate diagnosis, as seen in the second case.^1^

When blastomycosis was diagnosed in the 2 teenage patients, immunodeficiency was suspected but later ruled out. Although *Blastomycoses* can infect immunocompetent individuals, it is more reported in immunocompromised individuals who experience increased severity and mortality in immunocompromised patients, especially when diagnosis is delayed.^1^ Our work highlights the need to entertain this diagnosis in immunocompetent patients with consistent pulmonary and cutaneous clinical findings.

The rise in blastomycosis among immunocompetent individuals is unclear, although cannabis use has been suggested as a factor.^9^ In addition to contamination by species like cryptococcus, mucor, and aspergillus, smoking-induced structural and immunological damage to the lungs increases susceptibility to infection. Vitamin A deficiency, as seen in Case 1, also weakens immunity, making individuals more prone to opportunistic fungal infections.^10^

## Conclusion

A high degree of clinical suspicion and microbiologic data is needed to diagnose blastomycosis. Without timely treatment, primary disease will disseminate and become fatal. As cutaneous blastomycosis is the most common extrapulmonary manifestation of disseminated disease, skin examinations and tissue biopsies are critical for diagnosis. Immunocompetent individuals may present only with cutaneous disease, making prompt evaluation crucial. These cases illustrate the important role of hospital dermatologists in diagnosing blastomycosis in immunocompetent individuals.

## Conflicts of interest

None disclosed.
